# The Use of Immersive Virtual Reality in Reducing Anxiety Among Emergency Healthcare Professionals

**DOI:** 10.7759/cureus.104170

**Published:** 2026-02-24

**Authors:** Oriol Yuguero Torres, Itziar Lopez-Vena, César Pardos, Silvia Gros, Cecilia Llobet, Maria Viladrosa

**Affiliations:** 1 Research, E-RLab, Open University of Catalunya (UOC), Barcelona, ESP; 2 Research, ERLab, Institut de Recerca Biomèdica de Lleida (IRBLleida), Lleida, ESP; 3 Emergency Medicine, University Hospital Arnau de Vilanova, Catalan Health Institute, Lleida, ESP

**Keywords:** anxiety, burnout prevention, emergency professionals, immersive virtual reality, work-related stress

## Abstract

Introduction: Anxiety and burnout are common among emergency department (ED) healthcare professionals. Immersive virtual reality (IVR) may offer an on-demand, feasible strategy to support staff well-being in high-pressure settings. We evaluated whether an immersive projection-based IVR program was associated with changes in anxiety, burnout, perceived stress, and physiological stress markers in ED professionals.

Methods: We conducted a prospective within-subject, quasi-experimental study in a hospital ED. Of 198 eligible professionals, 131 enrolled. The study followed a fixed sequence: a four-week IVR phase (four weekly 20-minute sessions using the MK360 immersive projection system), an eight-week washout, and a four-week control phase (four 20-minute "usual rest" sessions). Emotional outcomes were assessed at baseline and end-of-phase for both IVR and control using the Goldberg Anxiety Scale (GAS; prior 15 days), Maslach Burnout Inventory-Human Services Survey (MBI-HSS), and Perceived Stress Scale-14 (PSS-14; prior month). Heart rate (HR) and blood pressure (BP) were measured immediately pre/post each session.

Results: Sixty-seven participants completed all sessions and assessments. The mean GAS decreased during the IVR phase (4.0±3.2 to 3.2±3.2; p<0.05), while changes in MBI-HSS subscales and PSS-14 were small and not statistically significant. At the end of the IVR phase, 25.4% reported feeling emotionally better, and none reported feeling worse. During the control phase, the mean GAS showed a smaller decrease (2.9±2.9 to 2.6±2.9), and 17.9% reported feeling better, while 4.5% reported feeling worse. Across IVR sessions, pre-post reductions were observed in HR and systolic BP in several sessions, whereas changes during usual rest were generally smaller and less consistent.

Conclusion: In this fixed-sequence, within-subject study, an immersive projection-based IVR program was associated with reduced self-reported anxiety and acute decreases in selected physiological stress markers among ED professionals. Findings should be interpreted in light of attrition and potential period effects; further controlled studies are warranted.

## Introduction

The burnout experienced by healthcare professionals has emerged as a silent epidemic in our country. Some data suggest that the prevalence of high burnout levels may be approximately 40%. This issue also extends to medical students, with 37% reporting significant burnout levels [1].

According to the 2022 report by the Galatea Foundation, 47.1% of medical professionals and 58.3% of nursing professionals are at risk of developing a mental health disorder. Notably, 26% of female healthcare workers considered seeking mental health services (compared to 11.6% in July 2020), while 16.1% of male healthcare workers did so (compared to 9% in July 2020) [2]. The report further indicates that 17% of healthcare professionals experience anxiety, alongside an increase in the consumption of tranquillizers and hypnotics compared to pre-pandemic levels.

These figures have escalated exponentially in recent years, with the COVID-19 pandemic merely exacerbating the issue. Indeed, previous studies have documented how burnout surged among various professional groups post-pandemic [3]. Some authors have linked heightened emotional involvement during COVID-19 to empathic stress and emotional exhaustion, which may contribute to burnout [4].

The management of burnout requires a multidisciplinary approach, with an important component related to healthcare professionals' working conditions. Additional individual- and organizational-level strategies may also support emotional well-being, including mindfulness-based approaches [5] and work schedule reorganization [6].

Immersive virtual reality (IVR) applications in healthcare, particularly in psychology, are expanding rapidly. A relevant feature of IVR is its ability to simulate natural environments in a controlled and repeatable manner. In environmental psychology, the Attention Restoration Theory (ART) proposes that exposure to natural settings can support attentional recovery and stress reduction. Evidence syntheses suggest that the restorative effects of nature exposure are present but modest and heterogeneous, with variability across study designs and outcomes [7]. Building on this rationale, there is growing evidence supporting IVR-based psychological interventions for patients [8], educational applications [9], and emerging work on stress reduction among healthcare professionals [10,11].

A study led by researchers from the Hospital del Mar Research Institute (IMIM) and the Center for Biomedical Network Research (CIBER) in Epidemiology and Public Health (CIBERESP), published in 2020 [12], revealed that nearly half of the healthcare professionals in our country were at high risk of developing a mental disorder following the initial waves of the COVID-19 pandemic. Against this backdrop, we conducted a single-site pilot to explore whether a brief, four-session IVR program is feasible and associated with short-term reductions in perceived stress and immediate post-session decreases in physiological arousal among emergency professionals. This work aims to address gaps identified by reviews, namely, the need for pragmatic protocols reporting both psychometric and physiological outcomes, alongside feasibility and tolerability data. Therefore, this study aims to determine whether virtual reality (VR) can mitigate anxiety and influence burnout levels among emergency healthcare professionals. 

In this single-site pilot, our primary objective was to assess whether a brief four-session IVR program is associated with a short-term reduction in anxiety symptoms (Goldberg Anxiety Scale (GAS)) among emergency department (ED) professionals. Secondary objectives were to evaluate changes in perceived stress (Perceived Stress Scale-14 (PSS-14)) and burnout (Maslach Burnout Inventory-Human Services Survey (MBI-HSS)) across phases, to quantify acute physiological responses (pre-/post-session heart rate (HR) and blood pressure (BP)), and to describe feasibility and tolerability (uptake, completion, and cybersickness).

## Materials and methods

Study design

We conducted a prospective, within-subject, quasi-experimental study with a fixed sequence of phases: a four-week IVR intervention period, an eight-week washout interval, and a four-week control period ("usual rest"). The order was not randomized or counterbalanced due to operational constraints and to minimize contamination of the usual rest condition. Given this pragmatic constraint, a secondary aim was to inform the feasibility of implementation in a real-world ED context (e.g., uptake, session completion, and operational deliverability).

Sample size and inclusion and exclusion criteria

The minimum sample size required was determined to detect a standardized difference between the mean changes of both groups of 0.5. With a 95% confidence level and an 80% power in a bilateral test, at least 63 participants were needed without accounting for dropouts. Assuming a maximum of 5% dropout, the sample size was increased to a minimum of 67 participants.

All clinical professionals (physicians, nurses, and auxiliary nursing care technicians) from the ED (198 professionals at the time of the study) were contacted via email. Professionals or students with a history of claustrophobia, vertigo, or dizziness, as immersive virtual therapy could be counterproductive, were excluded. Moreover, professionals with an inability to complete the study (total duration of 16 weeks, including the washout period) were also excluded. 

Data collection

Sociodemographic Variables

Age, gender, professional category, and years worked were collected at the start of the project.

Clinical Variables

History of depression and/or anxiety and whether participants were receiving current treatment for these conditions were collected prior to the participation. 

Measurement Scales/Emotional State

The research team obtained all necessary approvals to administer the tests to the participants.

MBI-HSS [13]: It is a 22-item, seven-level Likert scale assessing emotional exhaustion, depersonalization, and personal accomplishment. The validated Spanish version by Moreno Jiménez et al. [14] was used, as employed in previous studies.

PSS-14 [15]: It is a brief self-report scale consisting of four items assessing the extent to which individuals perceive their lives as unpredictable, uncontrollable, and overwhelming over the past month. It is also a 14-item self-report scale assessing perceived stress over the past month. Items are rated on a 5-point Likert scale (0-4), yielding a total score range of 0-56; higher scores indicate greater perceived stress [16].

GAS [17]: It is a nine-item dichotomous (yes/no) scale, where the first four questions are mandatory, while the remaining five are answered only if any of the initial responses are affirmative. Participants were asked about symptoms experienced over the past 15 days. The validated Spanish version by Montón et al. was used [18].

Outcome Assessment Schedule

Emotional outcomes were assessed at four time points: (1) baseline before the IVR phase, (2) end of the IVR phase, (3) baseline before the control phase, and (4) end of the control phase. Specifically, anxiety symptoms were measured with the GAS (15-day window), perceived stress with the PSS-14 (past month), and burnout with the MBI-HSS. These instruments were not administered before/after each 20-minute session. Physiological variables (HR and BP) were measured immediately pre- and post-session to capture acute responses. Following the final session, participants responded to an anchor question assessing perceived emotional change: "Please indicate if there has been any change in your emotional state since the beginning of the study period by selecting one of the following options: much worse (-3), significantly worse (-2), slightly worse (-1), no change (0), slightly better (1), significantly better (2), much better (3)".

Cardiovascular Variables

HR and BP were measured immediately before and after each intervention and control session using a sphygmomanometer. Respiratory rate was not included because it was not routinely or reliably measurable immediately pre-/post-session in our setting without additional instrumentation or observer-based counting, which would have increased workflow disruption and measurement variability.

Other Variables

Caffeine or tea consumption: During the control phase, participants reported whether they consumed coffee or tea during their rest break.

Adverse effects were recorded immediately after each IVR session using a brief cybersickness checklist covering nausea, dizziness, headache, and visual discomfort (yes/no; severity if present).

Psychometric outcomes (GAS, PSS-14, MBI-HSS) were assessed only at baseline and at the end of each phase, whereas physiological outcomes (HR, systolic blood pressure (SBP), diastolic blood pressure (DBP)) were assessed immediately pre/post each of the four sessions in each phase to capture acute responses.

Intervention

During the intervention phase, students completed a four-session MK360 program combining guided mindfulness with immersive 360° projection over four weeks (target dose: one 20-minute session per week). The intervention was delivered using the MK360 immersive projection system (BROOMX, Barcelona). Sessions were self-scheduled (any day/time) to accommodate classes and clinical rotations. Each session followed a standardized structure: (i) five minutes of guided mindfulness (audio-guided attention to breathing/present-moment awareness), followed by (ii) ~15 minutes of immersive 360° projection accompanied by ambient music. To minimize habituation and explore acceptability across different restorative environments, the 360° visual content varied each week: natural landscapes, beach/ocean scenes, abstract/psychedelic imagery, and an urban cityscape. The mindfulness component was the only guided audio; the projection segment used non-verbal ambient music.

Sessions could be completed individually or as a shared experience in small groups (a maximum of four professionals). In practice, approximately 90% of sessions were performed in groups. Instructions for participation (setup, safety, and mindfulness guidance) were provided via a QR code accessible at the MK360 site. After the session, participants recorded BP and HR again. Measurements were obtained using the same sphygmomanometer and following standardized on-screen instructions (seated position, cuff placement, and immediate recording pre-/post-session). While values were recorded by participants, the procedure was designed to minimize variability; nevertheless, self-recording may introduce measurement error, which we acknowledge as a limitation. Adverse effects were monitored using a brief cybersickness checklist.

Control activity

The control activity also lasted 20 minutes and took place in the staff rest area, where professionals could engage in their usual relaxation methods. Participants' vital signs were recorded before and after their break using designated logbooks. However, we did not systematically record the specific activities performed during the usual rest break.

An eight-week interval was maintained between the intervention and control phases to reduce potential carryover effects and to mitigate (though not eliminate) differences in ED workload between phases. The washout period coincided with the winter season for operational reasons. However, seasonal factors (e.g., holidays and seasonal mood variations) may have influenced stress-related outcomes and cannot be fully accounted for in this fixed-sequence design.

Statistical analysis

Participants were assigned a unique study ID to link phase-level surveys across the four assessment time points. Descriptive statistics are reported as mean±standard deviation (SD) for continuous variables and as frequencies (percentage) for categorical data. Baseline characteristics of completers and non-completers were compared using the χ² test for categorical variables and Student's t-test or the Mann-Whitney U test for continuous variables, as appropriate. Emotional outcomes (GAS, PSS-14, and MBI-HSS) were assessed at baseline and end-of-phase for both the IVR and control phases; within-phase changes were analyzed using paired tests. To compare whether change differed between phases (IVR vs. control), we analyzed participant-level change scores (end-of-phase minus baseline) using paired comparisons and/or linear mixed-effects models with a random intercept for participant and fixed effects for phase, time, and their interaction (phase×time). Physiological outcomes (HR, SBP, and DBP) were measured immediately before and after each session (four IVR sessions and four control sessions); pre-post changes were analyzed using repeated-measures approaches accounting for within-subject correlation (mixed-effects models including condition, time (pre/post), session number, and relevant interactions). Effect sizes are reported as mean differences with 95% confidence intervals, alongside exact p-values. Statistical significance was set at α=0.05. Mixed-effects models used all available observations under a missing-at-random assumption; no imputation was performed. Analyses were conducted using Stata v18.0 (StataCorp LLC, College Station, Texas, United States).

Ethical considerations

The study was approved by the Comité de Ética de Investigación con Medicamentos (CEIm) of Hospital Universitari Arnau de Vilanova (approval number: CEIC-2878). The research was conducted in accordance with the principles of the Declaration of Helsinki. The processing, communication, and transfer of participants' personal data complied with Organic Law 3/2018 on Personal Data Protection and the Guarantee of Digital Rights (LOPD-GDD 3/2018) and Regulation (EU) 2016/679 of the European Parliament and the Council of 27 April 2016. All participants provided informed consent before participation and retained the right to withdraw from the study at any time.

## Results

Of 198 ED professionals, 131 (66.1%) enrolled; 67 completed all required sessions and assessments. At baseline, completers and non-completers did not differ significantly in gender, years of professional experience, self-reported history of anxiety/depression, or baseline psychological measures (MBI-HSS subscales, PSS-14, and GAS). In contrast, completers were older (mean 43.1±12.1 vs. 36.9±11.3 years; p=0.0037), and completion varied by professional category (p<0.001), with higher completion among physicians and lower completion among nursing staff. The enrolled sample included representation across the main professional categories.

No significant differences were detected regarding prior anxiety or depression. Among enrolled participants, non-completers had a higher proportion of ongoing pharmacological treatment than completers (Table 1).

**Table 1:** Characteristics of healthcare professionals by participation status Baseline comparisons between participants with complete and incomplete follow-up were conducted using Welch's t-test for continuous variables with unequal variances and the Mann-Whitney U test for non-normally distributed data. For categorical comparisons, Pearson's chi-squared test was used; however, Fisher's exact test was employed whenever expected cell counts were below 5 (e.g., professional category) to ensure validity. TCAE: nursing care assistant (acronym in Spanish)

Variable	Total (N=131)	Complete participation (n=67)	Incomplete participation (n=64)	Test statistic	P-value
Age (years), mean±SD	40.1±12.1	43.1±12.1	36.9±11.3	U=1675.0	0.037
Female, n (%)	100 (76.3)	54 (80.6)	46 (71.9)	χ²=1.40	0.237
Professional category, n (%)					
Nursing	64 (48.9)	26 (38.8)	38 (59.4)	Fisher	0.043
Medicine	26 (19.9)	22 (32.8)	4 (6.3)		
Aux. nursing (TCAE)	37 (28.2)	19 (28.4)	18 (28.1)		
Other	4 (3.1)	0 (0)	4 (6.3)		
Years worked, n (%)					
<5 years	44 (33.6)	19 (28.4)	25 (39.1)	χ²=6.44	0.169
5-10 years	23 (17.6)	9 (13.4)	14 (21.9)		
11-15 years	14 (10.7)	6 (9)	8 (12.5)		
16-20 years	15 (11.5)	12 (17.9)	3 (4.7)		
>21 years	35 (26.7)	21 (31.3)	14 (21.9)		
Under treatment (yes), n (%)	7 (5.3)	1 (1.5)	6 (9.4)	Fisher	0.057

Among the participants (first column of Table 1; n=67), the mean age was 43.1 years, and 54 (80.6%) were women. A total of 26 (38.8%) were nursing professionals, 22 (32.8%) were from Medicine, and the remaining 28.4% were nursing care assistant professionals.Additionally, 31.1% of the participants reported having worked for more than 20 years, while 28.4% had worked for fewer than five years. Although completers were older, years worked did not differ significantly by participation status (Table 1). Age and professional experience are not perfectly collinear in this workforce, as years worked may be influenced by late entry into the profession, career transitions, or employment interruptions.

Regarding mental health history, 55 (82.1%) participants reported no prior history of anxiety or depression, 16.4% had experienced anxiety, and 1.5% had a history of depression. None of the participants reported having experienced both conditions. Furthermore, 1.5% of participants reported being under treatment for these disorders.

Regarding the administered scales, burnout levels measured using the MBI-HSS placed participants within the moderate burnout range across all subscales. Perceived stress levels (PSS-14) indicated a moderate level of stress. Finally, anxiety levels (GAS) showed an average score at the threshold considered indicative of anxiety.

Emotional outcomes**

During the IVR phase, the mean GAS decreased from 4.0 (SD 3.2) at baseline to 3.2 (SD 3.2) at end-of-phase (mean change -0.8) (p<0.05) as we can see in Table 2.

**Table 2:** Descriptive analysis of study participants across both phases Longitudinal changes within groups (pre vs. post) were assessed using paired-samples t-tests. MBI-HSS: Maslach Burnout Inventory-Human Services Survey; PSS-14: Perceived Stress Scale-14; GAS: Goldberg Anxiety Scale

Outcome	Immersive reality: phase 1 initial	Immersive reality: phase 1 final	Test statistic	P-value	Control: phase 2 initial	Control: phase 2 final	Test statistic	P-value
MBI-HSS								
Emotional exhaustion	19.6±11.3	19.5±10.7	t=0.15	0.884	18.1±10.2	18.4±11.0	t=-0.37	0.710
Depersonalization	7.7±6.6	6.7±5.6	t=2.45	0.017	6.6±5.5	6.6±6.0	t=0.00	1.000
Personal accomplishment	37.0±7.8	37.9±6.4	t=-1.45	0.151	38.1±7.3	38.3±6.6	t=-0.42	0.675
PSS-14 (stress)	20.9±8.9	19.5±8.7	t=2.21	0.031	18.9±9.0	19.0±9.1	t=-0.19	0.852
GAS (anxiety)	4.0±3.2	3.2±3.2	t=2.97	0.004	2.9±2.9	2.6±2.9	t=1.34	0.185

The mean PSS-14 decreased from 20.9 (SD 8.9) to 19.5 (SD 8.7) (mean change -1.4), while MBI-HSS subscales showed minimal changes (Emotional Exhaustion: 19.6 to 19.5; Depersonalization: 7.7 to 6.7; Personal Accomplishment: 37.0 to 37.9).

During the control phase, the mean GAS decreased from 2.9 (SD 2.9) to 2.6 (SD 2.9) (mean change -0.3), PSS-14 remained essentially unchanged (18.9 to 19.0), and MBI-HSS subscales were stable (Emotional Exhaustion: 18.1 to 18.4; Depersonalization: 6.6 to 6.6; Personal Accomplishment: 38.1 to 38.3).

After the IVR phase, 25.4% reported feeling emotionally better and 0% worse; after the control phase, 17.9% reported feeling better and 4.5% worse as we can see in Table 3.

**Table 3:** Perceived emotional changes at the end of intervention (categorical) Due to the low frequency of responses in specific categories, differences in perceived emotional changes were analyzed using Fisher's exact test.

Response	Immersive reality: phase 1, n (%)	Control: phase 2, n (%)	Test	P-value
No changes	50 (74.6)	52 (77.6)	Fisher	0.489
Slightly better	13 (19.4)	8 (11.9)		
Much better	4 (6)	4 (6)		
Slightly worse	0 (0)	1 (1.5)		
Much worse	0 (0)	2 (3)		

Table 4 presents the vital signs recorded during the four sessions of the IVR intervention, measured both before (pre) and after (post) the intervention. The values remained within the thresholds considered normal for vital signs. The data indicated that post-intervention measurements were lower than pre-intervention values. These within-session differences were statistically significant for HR and SBP across all sessions and for DBP in the third session.

**Table 4:** Descriptive analysis of vital signs recorded during each session of the IVR intervention (phase 1) Descriptive analysis of vital signs collected during each session of the IVR intervention (phase 1), measured before (pre) and after (post) the intervention. Values represent the mean (SD). IVR: immersive virtual reality; HR: heart rate in beats per minute; SBP: systolic blood pressure in mmHg; DBP: diastolic blood pressure in mmHg *p<0.05 when comparing pre- and post-intervention values using the t-test; – no valid values

	HR pre	HR post	SBP pre	SBP post	DBP pre	DBP post
Session 1	77.4 (10.5)	74.1 (9.2)*	119.1 (15.8)	116.5 (14.2)*	78.3 (9.4)	77.4 (8.5)
Session 2	77.3 (10.1)	77.3 (10.1)	116.7 (14.0)	113.6 (11.9)*	76.3 (8.8)	74.7 (9.3)
Session 3	75.3 (10.2)	72.6 (8.9)*	117.3 (12.8)	113.0 (11.5)*	77.8 (8.4)	74.4 (12.7)*
Session 4	76.1 (9.7)	73.9 (9.5)*	116.1 (11.3)	113.7 (13.2)*	76.2 (6.6)	74.9 (8.0)

Table 5 presents the vital signs recorded during the four sessions of the rest intervention, measured both before (pre) and after (post) the intervention. The values remained within the thresholds considered normal for vital signs. The data indicate that post-intervention measurements were very similar to pre-intervention values, with some instances where post-intervention values were slightly higher.

**Table 5:** Descriptive analysis of vital signs recorded during each session of the rest intervention (phase 2) Descriptive analysis of vital signs collected during each session of the rest intervention (phase 2), measured before (pre) and after (post) the intervention. Values represent the mean (SD). HR: heart rate in beats per minute; SBP: systolic blood pressure in mmHg; DBP: diastolic blood pressure in mmHg *p<0.05 when comparing pre- and post-intervention values using the t-test; – no valid values

	HR pre	HR post	SBP pre	SBP post	DBP pre	DBP post
Session 1	76.3 (10.2)	76.1 (10.7)	114.9 (13.3)	116.7 (18.3)	75.4 (9.8)	73.9 (12.5)
Session 2	74.3 (12.6)	74.5 (10.1)	116.2 (12.2)	115.3 (13.2)	75,6 (8.0)	73.8 (8.1)*
Session 3	75.6 (10.5)	76.1 (10.2)	115.9 (13.8)	115.6 (13.2)	76.1 (8.3)	74.8 (9.2)
Session 4	75.5 (10.2)	75.5 (9.8)	115.6 (13.0)	115.2 (13.6)	75.5 (10.2)	73.9 (13.9)

Among all measurements, DBP showed the most evident reduction in post-intervention recordings, with the difference reaching statistical significance only in the second session.

When comparing differences in vital signs across sessions, post-intervention values did not always decrease following the rest intervention. Instead, they either remained stable or were slightly higher after the intervention. Only DBP showed a downward trend in post-intervention measurements.

Finally, more than half of the participants consumed stimulants (coffee and/or tea) during the rest period (Table 6).

**Table 6:** Descriptive analysis of the percentage of professionals who consumed stimulants (coffee and/or tea) during the rest session (phase 2) The values shown represent the percentage (frequency) of professionals who consumed stimulants (coffee and/or tea) during the rest session (phase 2).

	Consumption of stimulants (coffee and/or tea)
Session 1	55.2% (37)
Session 2	58.2% (39)
Session 3	56.7% (38)
Session 4	59.7% (40)

Tolerability was high. No serious adverse events were reported. Cybersickness symptoms were absent or mild across sessions, and no participant discontinued a session due to cybersickness.

## Discussion

IVR significantly reduces anxiety levels among emergency professionals compared to their usual rest periods. Additionally, it significantly lowers HR and BP. These findings are consistent with prior workplace applications of VR relaxation in healthcare staff, which report acute reductions in stress and improved affect following brief, on-shift VR "mental breaks" [19,20]. Regarding emotional well-being, although 74.6% of participants reported no noticeable change, 25.4% stated that they felt better. A significant reduction in the GAS score (p<0.05) was observed, alongside a decrease in burnout and perceived stress levels, although these reductions did not reach statistical significance. This pattern is plausible because acute anxiety and arousal markers may be more responsive to brief interventions than broader, more stable constructs such as burnout, as suggested by systematic reviews of VR-based stress management and relaxation interventions [21]. Tolerability was favorable, with few and mostly mild cybersickness symptoms. This is relevant for implementation in ED settings, where brief interventions must not impair clinicians' functioning during shifts. Future studies should standardize adverse event reporting and examine whether susceptibility differs by prior VR exposure.

A recent review evaluated various strategies aimed at improving healthcare professionals' well-being and highlighted that workplace interventions tend to show modest, short-term benefits while also emphasizing operational barriers such as workload and time constraints [22]. Our experience aligns with these observations. Despite broad invitation across the ED, completion was moderate, and retention differed by professional category, which is compatible with workload and scheduling constraints limiting sustained participation, particularly among nursing staff. As a pragmatic adaptation, we reduced the number of sessions to improve adherence, highlighting the need for protected time, staffing support, and a dedicated space to facilitate implementation in future pragmatic trials. Moreover, another review [23] evaluated various strategies aimed at improving healthcare professionals' well-being. Other initiatives, such as mindfulness-based programs, have also been shown to improve stress-related outcomes among healthcare workers; however, implementation in hospital settings is often challenged by factors such as staffing pressures, limited protected time, and the need to fit interventions into complex workflows [24].

Our intervention was fully autonomous and could be undertaken on demand during the day. Sessions could be delivered individually or in small groups (up to four participants); in practice, most sessions in our study were group-based (~90%), which may enhance scalability but could also influence the experience through social/contextual effects. This is important because it allows professionals to manage their own time and decide when the intervention would be most beneficial for them. In line with this, VR-based guided meditation and brief VR meditation programs have been described as feasible and accessible mindfulness-based options for healthcare workers that can be delivered in the workplace with flexibility around scheduling [25].

To assess whether IVR provided an added benefit to the workplace environment, we compared its effects to those of a standard rest period, which typically involved discussions with colleagues, social media browsing, or checking personal emails. To assess whether IVR provided an added benefit, we compared its effects with a usual rest period. Specific activities during the control breaks were not systematically documented; however, given the setting and time constraints, usual breaks in the ED often consist of unstructured activities (e.g., social interaction or passive smartphone use). Evaluations of well-being during the usual rest period showed no reduction in stress levels. In some cases, burnout and perceived stress scores even increased. When asked about perceived emotional impact, a small proportion of professionals reported feeling better during the usual rest period, while very few reported feeling worse (Table 3). These descriptive responses should be interpreted cautiously, as formal symptom scales did not show statistically significant changes. Nonetheless, the pattern is hypothesis-generating and suggests that unstructured breaks may not be uniformly restorative in high-demand environments; future studies should better characterize break activities and test whether more structured relaxation cues yield more consistent short-term downregulation of arousal.

Regarding vital signs, changes during the usual rest period were small and generally not statistically or clinically meaningful. Although an isolated finding reached nominal statistical significance (DBP decreased), this should be interpreted cautiously given the number of comparisons performed and the potential for chance findings. Caffeine consumption during breaks was common and may have contributed to variability in physiological measures. We included pre/post vital signs as an objective indicator of acute physiological downregulation. In our study, IVR sessions were associated with consistent pre-post reductions in HR and SBP, supporting an acute relaxation response and aligning with prior reports on VR-based relaxation interventions [26]. In contrast, changes during usual rest were small and generally not clinically meaningful.

It is also noteworthy that professionals who did not complete the study exhibited higher burnout levels than those who participated. Prior evidence links burnout and perceived resource loss to less favorable attitudes toward organizational change, which may plausibly translate into reduced engagement with optional workplace interventions under time pressure [27]. Consequently, IVR could be particularly useful as a low-threshold supportive or preventive tool for individuals experiencing mild-to-moderate distress, while additional implementation strategies may be needed to reach and retain those with higher baseline burden. Differential completion by age and professional category suggests the possibility of attrition-related selection effects. However, baseline burnout (MBI-HSS), perceived stress (PSS-14), and anxiety (GAS) did not differ significantly between completers and non-completers, which reduces the likelihood that the observed effects reflect a "healthier" subgroup with lower initial distress. Nonetheless, the higher completion among physicians and lower completion among nursing staff may limit generalizability across professional groups and should be addressed in future pragmatic trials through tailored implementation strategies. 

The higher proportion of ongoing pharmacological treatment among non-completers may suggest differential participation related to mental health status or time/energy constraints, which could contribute to selection bias. However, baseline symptom scores (MBI-HSS, PSS-14, GAS) were similar between completers and non-completers.

The primary limitation of our study was participant attrition throughout the study period. Although we achieved the target sample size, some participants dropped out. To improve adherence, we reduced the number of sessions. This attrition may limit generalizability and could introduce selection bias if completion was influenced by time availability, workload, or baseline mental health. However, baseline burnout (MBI-HSS), perceived stress (PSS-14), and anxiety (GAS) did not differ significantly between completers and non-completers (Table 1), which reduces the likelihood that completers were systematically less distressed at baseline. Importantly, we did not collect baseline measures from eligible professionals who did not enroll; therefore, we cannot determine whether non-participants differed systematically from participants or represented a more distressed subgroup that might have derived greater benefit. Additionally, the assessment scales used were self-reported, meaning participants themselves rated their emotional state. To mitigate this limitation, we included physiological markers as objective indicators of changes following the intervention. The fixed-order design (IVR first, control later) introduces potential period/season/workload and expectancy effects; therefore, causal inference regarding between-condition differences should be interpreted cautiously. Pre/post cardiovascular measures were self-recorded, which may introduce reporting or measurement bias despite standardized procedures. Because the control condition was unstructured and participants were aware of being observed, a Hawthorne effect cannot be excluded; usual rest behaviors and self-reports may have differed from routine practice.

Despite these limitations, the pragmatic design allowed us to observe feasibility-relevant aspects. Implementation of IVR sessions within the ED workflow appeared operationally deliverable: the intervention was structured in brief 20-minute sessions and was often delivered in small groups, which may increase scalability. However, overall completion rates were moderate and suggest logistical and workforce barriers that should be addressed in future trials (e.g., protected time, staffing, and dedicated space). Formal implementation outcomes (acceptability, appropriateness, fidelity, and cost) were not systematically measured and should be incorporated in subsequent pragmatic randomized controlled trials (RCTs).

To ensure our sample was as representative as possible, all professionals within the ED were invited to participate. As a result, our study sample included approximately 30% representation from each professional group.

## Conclusions

IVR was associated with a statistically significant short-term reduction in anxiety symptoms among ED professionals and with consistent post-session decreases in HR and SBP, supporting an acute relaxation response. In contrast, changes in perceived stress and burnout were small and did not reach statistical significance over the study period. Importantly, the intervention was well tolerated (no cybersickness symptoms reported) and operationally deliverable as brief, on-shift sessions, although completion was moderate and differed by professional category, highlighting the need for protected time and implementation strategies to ensure reach across staff groups. This is, to our knowledge, the first study in our country to evaluate IVR across the main professional categories in an ED, and it adds feasibility and tolerability data to the still-limited evidence base in emergency care settings.

These findings support further research using robust designs with longer follow-up, strategies to improve adherence across professional groups, and outcomes that capture both immediate and sustained effects. From an implementation perspective, hospitals should consider providing protected time and dedicated quiet spaces that facilitate brief, on-demand recovery interventions during shifts, particularly in high-pressure environments such as EDs. Nevertheless, IVR should be viewed as a supportive or preventive tool rather than a stand-alone solution: meaningful reductions in burnout will require multifactorial interventions addressing organizational and system-level drivers, with active engagement from healthcare management and clinical leadership.

Beyond the observed changes in anxiety, our findings also provide preliminary, real-world information on the operational deliverability of brief IVR sessions in an ED setting; future work should formally evaluate implementation outcomes and cost-effectiveness.
